# Brain regions involved in observing and trying to interpret dog behaviour

**DOI:** 10.1371/journal.pone.0182721

**Published:** 2017-09-20

**Authors:** Charlotte Desmet, Alko van der Wiel, Marcel Brass

**Affiliations:** 1 Department of Experimental Psychology, Ghent University, Ghent, Belgium; 2 Ghent Institute for Functional and Metabolic Imaging of the brain, Ghent University, Ghent, Belgium; 3 Department of Work and Organisation Studies, Faculty of Economics and Business, KU Leuven, Louvain, Belgium; University College London, UNITED KINGDOM

## Abstract

Humans and dogs have interacted for millennia. As a result, humans (and especially dog owners) sometimes try to interpret dog behaviour. While there is extensive research on the brain regions that are involved in mentalizing about other peoples’ behaviour, surprisingly little is known of whether we use these same brain regions to mentalize about animal behaviour. In this fMRI study we investigate whether brain regions involved in mentalizing about human behaviour are also engaged when observing dog behaviour. Here we show that these brain regions are more engaged when observing dog behaviour that is difficult to interpret compared to dog behaviour that is easy to interpret. Interestingly, these results were not only obtained when participants were instructed to infer reasons for the behaviour but also when they passively viewed the behaviour, indicating that these brain regions are activated by spontaneous mentalizing processes.

## Introduction

"The dog is man's best friend.

He has a tail on one end.

Up in front he has teeth.

And four legs underneath."

(from 'An introduction to dogs' by Ogden Nash, 1920)

The first line of this poem has been quoted many times and reflects what a lot of dog owners will agree upon: their dog is their best friend. The tight relationship between humans and dogs has its origins thousands of years ago when wild wolves were trained to assist in hunting, herding cattle or to protect against enemies. Depending on the breed, dogs can fulfill multiple functions such as guide dogs, watchdogs or mere companions. Research has shown that the relationship between dog owners and their pets influence the social and psychological well-being of the dog owner and might even relate to physical health benefits (e.g. [1]). Human-dog interactions can thus impact our lives extensively.

From this perspective the question arises what characterizes this tight social relationship between humans and dogs. Do we use the same neuro-cognitive mechanisms when socially interacting with humans and dogs? In particular, we are interested in the question whether mentalizing about dog behaviour activates the same brain regions than mentalizing about human behaviour. Although there have been studies examining brain activity during the observation of dog behaviour, the brain mechanisms involved in mentalizing about dog behaviour have never been systematically investigated.

fMRI studies investigating brain mechanisms engaged by the *observation* of dog behaviour, showed activation in the mirror neuron network [2] when the observed behaviour was in the behavioural repertoire of the observer. For example, observing an eating dog activated the mirror neuron network, while a barking dog did not. Furthermore, Kujala and colleagues showed stronger activation of several brain regions in dog experts when watching pictures of dogs facing each other compared to pictures of dogs looking away from each other [3]. This pattern was absent for non-experts. In addition, the magnitude of brain activation in the pSTS was correlated with the expertise one has with dogs. Dog expertise thus seems to be correlated with the amount of brain activation when observing dog behaviour. Interestingly, a study on the behavioural level showed that dog owners strongly mentalize when observing dog behaviour. Horowitz (2009) conducted a study in which dogs were offered a forbidden treat in the absence of their owners. The owners, were informed that their dog was ‘‘guilty” or that the dog behaved well (this information could be right or wrong). Owners were then reunited with their dogs and either scolded or greeted their dog (based on the information that was given). The results indicated that the interpretation of dogs’ facial expressions (guilty expression) were more indicative of the behaviour of the owner (did the owner scold at the dog or not) than of the behaviour of the dog (was the scolding justified). One could thus assume that dog owners will engage a similar network when interpreting dog behaviour as when interpreting human behaviour compared to nonowners [4].

However, to our knowledge there are no studies examining the brain regions involved in mentalizing about dog behaviour. This is surprising since the brain correlates of mentalizing in human-human interaction has been studied extensively. The ability to infer intentions or mental states from other humans has been termed mentalizing or theory of mind (ToM) [5]. Mentalizing has been related to a network in the brain consisting of the medial prefrontal cortex, bilateral temporo-parietal junctions and posterior cingulate cortex (e.g. [6, 7]). Typically, the ToM network is more engaged when humans observe behaviour that is difficult to interpret compared to when humans observe behaviour that is easy to interpret (e.g. [8, 9]); but see also [10]). Further, it seems that this brain network is responsive when humans are instructed to infer underlying reasons for an observed behaviour (e.g. [11]). Importantly, activation of the ToM network was also observed when humans passively observe other's behaviour (e.g. [12, 13]).

In the current study we address three questions. First, is brain activation in the ‘mentalizing system’ stronger when observing dog behaviour that is difficult to interpret compared with observation of dog behaviour that is easy to interpret? Second, is this potential difference in brain activation amplified when participants are explicitly instructed to reason about dog behaviour compared to passive observation? Finally, does the activation pattern depend on how much experience participants have with dogs?

We think that addressing these questions will facilitate our understanding of how humans interact with dogs but will also facilitate our understanding of the human ‘mentalizing’ system. Regarding the first point, we will learn whether human-dog interaction is based on similar neuro-cognitive mechanisms as human-human interaction. Regarding the second point, we will learn more about the limits of the human mentalizing system: Is the mentalizing system restricted to the interpretation of human behaviour or will it also respond to the observation of other non-human intentional agents such as dogs? Finally, by looking at expertise with dogs, we will be able to determine whether involving the mentalizing network when observing dog behaviour is something everybody is doing or is restricted to ‘experts’?

To answer the above questions we first determined the network involved in mentalizing using an established localizer task. Then we investigated whether brain areas identified in this localizer task are also responsive when humans try to interpret dog behaviour. To this aim, we let participants observe video clips showing dog behaviour. Some video clips showed dog behaviour that was easy to interpret (for example a dog drinking water) while other video clips showed dog behaviour that was difficult to interpret (for example a dog eating grass). We then examined if the mentalizing network was more engaged during the observation of video clips that were difficult to interpret compared to the observation of video clips that were easy to interpret. To investigate whether these areas were only responsive when participants explicitly mentalized about the dog behaviour, participants observed the video clips of the dog behaviour in two different tasks. During the first task (PASSIVE), participants passively observed the video clips. During the second task (INTERPRETATION), participants were explicitly instructed to infer the reasons for the dog behaviour. If the processes underlying ToM are spontaneous, then we expect activation of the localizer network during both tasks. Moreover, we then expect more engagement of the localizer areas for video clips that are difficult to interpret compared to video clips that are easy to interpret during both tasks. If, on the other hand, the localizer network is only activated when participants are explicitly instructed to infer reasons for the dog behaviour, we expect a difference in engagement between difficult and easy video clips in the INTERPRETATION task but not in the PASSIVE task. Finally, we examined if dog ownership modulates activation in the localizer network and if this is different for both tasks (PASSIVE and INTERPRETATION).

## Materials and methods

### Participants

18 subjects participated in the experiment. One participant stopped the experiment prematurely and was excluded for further analysis. From the remaining 17 participants (11 females, mean age = 23 years, SD = 3 years), 8 participants were dog owners and 9 participants did not have a dog. All participants had normal or corrected-to-normal vision, were right handed as measured by the Edinburgh Handedness Inventory [14] and had no history of neurological disorders. Participants received 33 euro in turn for participation. The study was approved by the Medical Ethical Review Board of the Ghent University hospital (approval number: B670201317953). Before participants took part in the study they gave written consent to participate. The consent procedure was approved by the Ethical Review Board.

### Stimuli and design

The experiment was programmed with presentation software (Neurobehavioral Systems, Albany, NY). Three tasks were offered to the participants. During the first two tasks 40 different video clips of dog behaviour were shown. The videos differed in how easy it was to interpret them. Some videos comprised dog behaviour that was rather easy to interpret (for example a dog drinking water) while other videos comprised behaviour that was more difficult to interpret (for example a dog eating grass). The classification of video clips as ‘easy to interpret’ or ‘difficult to interpret’ was made based on ratings participants gave after scanning. After scanning, participants saw every video clip again and had to indicate on a scale from 1 (not difficult at all) to 10 (very difficult) how difficult it was to interpret the dog behaviour. A mean score of these ratings was computed for every video clip. The ten video clips with the highest scores were classified as ‘difficult to interpret’, the ten video clips with the lowest score were classified as ‘easy to interpret’. The dogs that were shown in the videos consisted mainly of three dachshunds. Other breeds that were shown in the videos were a German shepherd, a Siberian Husky and several cross breeds. The mean duration of the videos was 5.8 seconds (SD = 0.72 seconds with a range from 4 to 7 seconds). The clip duration did not differ between the video clips that were ‘easy’ and ‘difficult’ to interpret (mean duration of the ‘easy’ videos = 6.00 seconds, mean duration of the ‘difficult videos = 5.50 seconds, *t*(18) = 1.63, *p* = 0.12).

During the final task (the ToM localizer task) 8 different videos were shown. These videos were the original random (RANDOM) and theory of mind (ToM) animations used by Castelli and colleagues [15]. The videos were silent animations of two triangles (one small blue triangle and one larger red triangle). During the RANDOM videos the triangles did not interact and showed movements that were not goal directed (for example the two triangles were bouncing around). During the ToM videos, the triangles interacted in such a way that they implied mental states (for example one triangle was seducing the other). The video clips were on average 39 seconds (SD = 2 seconds, with a range from 35 seconds to 41 seconds). For a description of the video clips presented in the first two tasks see Table 1.

**Table 1:** Description of the 40 video clips showing dog behaviour and the corresponding possible interpretation(s). The twenty video clips that received the most extreme scores on the difficulty ratings after scanning are marked with a cross in the column ‘easy’ (10 clips with the lowest scores) or ‘difficult’ (10 clips with the highest scores). In the fMRI analyses, brain activation during the observation of these ten difficult video clips is compared with brain activation during the observation of these ten easy video clips. Some video clips showed the same behaviour. For example, there are two video clips that showed a dog drinking water. When this is the case the number of video clips showing this behaviour is reported between brackets.

	possible interpretation(s)	easy	difficult
**Description of the behaviour shown in the video clips**			
rubbing the neck against the ground	scratching put a smell from the ground onto himself		x
placing the mouth over the mouth of another dog	showing dominance		
eating leafs	to promote digestion		
making a hole in the ground	digging, searching something		x
wiggling the tail and looking up	the dog expects something from the person in front of him		
smelling at a tree and marking	the dog marks the tree		
scratching on a blanket (2)	creating a place to sleep		
turning in a circle and laying down	creating a place to sleep		
walking and suddenly freezing	orienting response before going after something		
scratching at the end of the couch	dog is afraid to jump off the couch		x
turning on the back	scratching		x
covering something with earth	after urinating, covering it with earth		x
two dogs look at something, then one attacks the other	something interesting catches the attention of the dogs, then the attacking dog claims it		x
standing up and sniffing	there is something that is held above the dog that the dog wants	x	
running away, tail is first upright and then it goes down	first the dog has a dominant pose, then the dog walks away		x
laying before a nut and then eating it	the dog waits for a command to eat the nut		
sitting up straight and still	the dog hears something		
eating grass	to promote digestion		
licking the ground	the dog has smelled something and then licks		x
two dogs sniffing at each other's back	to meet each other		
licking at the paw	Washing		
eating dry dog food	Eating	x	
chewing at a bone	Chewing	x	
drinking water (2)	Drinking	x	
scratching (2)	Scratching	x	
sniffing at grass	Smelling		x
shaking out	shaking out		
sneezing and then showing the teeth	sneezing and then showing dominance		x
two dogs smelling at each other's noses	to meet each other		
Sleeping	Sleeping	x	
stretching the corps	stretching after sleeping		
Barking	barking because something interesting has happened		
walking at the leash and yawning	walking and yawning (because of fatigue or stress)		
lying in basket and yawning	yawning because the dog is tired	x	
licking the back	Washing	x	
tearing at the leash	the dog does not want to walk		
a dog is lying down and wheezes	resting after an effort		

### Procedure

Three functional fMRI runs were presented. The three runs were always presented in the same order and consisted of the following three tasks: PASSIVE task, INTERPRETATION task and LOCALIZER task.

The first task (PASSIVE) showed the abovementioned videos of dog behaviour. Participants were instructed to watch the videos attentively. On 20% of the videos a red square would appear at the bottom left of the screen. Participants were instructed to press with their right index finger when they detected a red square. We gave participants this additional task to ensure that they indeed watched the videos. Before presentation of the video clip, a fixation cross appeared on screen for 200 ms. Between two videos a blank screen was shown during a jittered interval. The duration of the interval was computed by applying a pseudo-logarithmic function. Half of the inter-trial intervals were short (range between 200–2000 ms in steps of 600 ms), one third of the intervals were intermediate (range between 2600 ms and 4400 ms) and one sixth of the intervals were long (range between 5000 ms and 6800 ms) with a mean intertrial interval of 2700 ms. In this first task, the videos were presented in two blocks with this restriction that the last video of the first block and the first video of the second block were not the same. The order of video clips was randomized within each block. The PASSIVE task, or the first fMRI run, consisted of 80 trials (40 videos x 2 blocks) and lasted about 15 minutes.

During the second task (INTERPRETATION), participants saw the same videos as during the PASSIVE task. However, this time participants were instructed to think about what the dog in the video was doing, and why that dog was doing so. These instructions were given both verbally and on screen just before the participant started the second task. Before the presentation of the video a fixation cross appeared during 200 ms. After each video participants had to indicate on a 4 point-scale how difficult they thought the behaviour in the video was to interpret. The four options were listed onscreen from left to right and comprised the following options: ‘very easy’, 'easy', 'difficult' and ‘very difficult’. They had to respond by pressing one of the four corresponding response buttons. A randomized jitter interval, computed in the same way as in the PASSIVE task, was inserted before and after each video. As in the passive task, the video clips were presented in two blocks resulting in 80 trials (40 video x 2 blocks) with a randomized order of videos within each block. This second fMRI run lasted about 20 to 25 minutes, depending on the participants' performances.

Participants responded by means of two response boxes containing two response buttons each. The two boxes were placed on their left and right upper leg. The left index and middle finger were placed on the left button box and the right index and middle finger on the right button box.

During the third and final task (LOCALIZER) animations of a couple of triangles were shown. Participants were instructed that the two triangles would interact with each other and that they had to think about what was happening in the animation. They were also informed that after scanning they would have to recite what they thought happened in the video. Before the onset of the video clip a fixation cross was presented during 200 ms. In addition to the videos we presented blank screens that lasted 5 seconds. In total 8 blank screens were shown and these were randomly intermixed with the video clips. As in the previous two tasks, the videos were presented in two blocks with the additional restriction that the last video of the first block was not immediately repeated in the second block. Within each block the order of ToM and RANDOM videos was randomized. The LOCALIZER task consisted of 16 trials (8 videos x 2 blocks). The timing between two videos was jittered in the same pseudologarithmic way as described for the two previous tasks. This final fMRI run lasted about 15 minutes.

Finally after scanning, all videos were shown again. First, participants saw all videos containing the dog behaviour. The video clips were shown in a randomized order and after each video, participants had to recite what they thought the dog was doing and why the dog was doing so. They also had to indicate how difficult they felt the behaviour was to interpret, on a more sensitive scale ranging from 1 to 10 (“1” being extremely easy and “10” being extremely difficult). Their answers were given vocally and recorded using Audacity software. Second, the videos containing the animation of the triangles were shown. Here again, participants had to recite what they thought the triangles were doing and why they were doing so. They also had to indicate on a scale ranging from 1 to 10 how difficult they thought the behaviour was to interpret.

### fMRI methods

The experiment was conducted on a 3 T scanner (Siemens Trio) using a 32 channel head coil. Participants were placed head first and supine in the scanner. Before the actual experiment started, 176 high resolution anatomical images were acquired using a T1-weighted 3D MPRAGE sequence [repetition time (TR) = 2530 ms, echo time (TE) = 2.58 ms, image matrix = 256 × 256, field of view (FOV) = 220 mm, flip angle = 78, slice thickness = 0.90 mm, voxel size = 0.9 × 0.86 × 0.86 mm(resized to 1 × 1 × 1 mm)]. During the experiment whole-brain functional images were obtained which were acquired using a T2*-weighted echo planar imaging (EPI) sequence, sensitive to BOLD contrast (TR = 2000 ms, TE = 28 ms, image matrix = 64 × 64, FOV = 224 mm, flip angle = 80°, slice thickness = 3.0 mm, distance factor = 17%, voxel size 3.5 × 3.5 × 3 mm, 34 axial slices).

### fMRI preprocessing

All data were analyzed with SPM8 (Wellcome Department of Imaging Neuroscience, UCL, London, U.K.; www.fil.ion.ucl.ac.uk/spm). In order to account for T1 relaxation effects, the first four scans of the three runs were considered dummy scans. All functional images were spatially realigned using a rigid body transformation. Then, they were slice-time corrected with respect to the middle acquired slice. Next, the structural image of each subject was co-registered with their mean functional image. During segmentation, the structural scans were brought in line with the tissue probability maps available in SPM. The parameters estimated during the segmentation step were then used to normalize the functional images to standard MNI space. Finally, the images were resampled into 3 mm^3^ voxels and spatially smoothed with a Gaussian kernel of 8 mm (full-width at half maximum). A high-pass filter of 128 Hz was applied during fMRI data analysis.

### fMRI LOCALIZER analysis

The data of the LOCALIZER task were analyzed by means of a mixed model with a standard two stage procedure [16]. At both stages the analysis was performed using the general linear model as implemented by SPM8. During the first stage we defined two regressors of interest representing the type of video that was shown (ToM or RANDOM). The duration of the entire video was modeled and convolved with the canonical hemodynamic response function (HRF). To account for movement artefacts we entered six additional motion regressors into the model. In addition, the blank presentations of 5 seconds were entered as regressors of no interest. At this first level of analysis, contrast images were created for each subject by comparing the parameter estimates for the canonical HRF. Contrasts were made for the following effect: ToM > RANDOM. At the second stage, this contrast image was entered into a random effects analysis with subject as a random variable. Group contrast images were generated by one sample *t*-tests. To correct for multiple comparisons a cluster-extent based thresholding approach was used [17]. First, a primary uncorrected threshold of *p* < 0.001 at voxel level was used to identify groups of suprathreshold voxels. Second, a cluster-level extent threshold, represented in units of contiguous voxels (k), was determined by SPM 8 (*p* < 0.05 FWE cluster corrected threshold). Only clusters that have a k value that is equal or larger than this threshold are reported.

### fMRI task analyses

We wanted to investigate whether the regions engaged during the localizer task, are also engaged when trying to interpret the dog behaviour. In order to classify the video clips as difficult or easy to interpret, we used the ratings participants gave after scanning. As previously mentioned, participants saw all videos containing dog behaviour again when they left the scanner and had to indicate how difficult it was to interpret the dog behaviour. We computed mean scores for each video clip and then selected the ten video clips that had the highest mean scores (mean score = 5.8/10, SD = 0.56) as DIFFICULT videos and the video clips that had the lowest mean scores (mean score = 2.1/10, SD = 0.73) as EASY videos.

These EASY and DIFFICULT video clips were then entered as two regressors in two general linear models (one for the PASSIVE task and one for the INTERPRETATION task) on the subject level. The duration of the entire video clip was modeled and convolved with the canonical hemodynamic response function (HRF). Movement parameters were included in the regression model to account for variance associated with head motion. Mean beta's for the events of interest (EASY and DIFFICULT video clips) were extracted for the PASSIVE and the INTERPRETATION task using the MARSBAR toolbox for SPM in the significant clusters found in the localizer task [18]. The beta-values obtained were then subjected into a repeated measures ANOVA containing the factor DIFFICULTY (easy versus difficult) as a within subjects factor and the factor DOG OWNER (dog owner versus non-owner) as a between subjects factor both for the PASSIVE and the INTERPRETATION task.

## Results

### Localizer task

As in [15], the contrast ToM > RANDOM was computed to examine brain areas that are more engaged during mentalizing than during observing random movements. This contrast revealed 4 significant clusters. The left superior temporal sulcus (lSTS, -60; -55; 7), the right superior temporal sulcus extending into the right temporo-parietal junction (rTPJ, 57; -55; 1) and two clusters in the right inferior frontal gyrus (one in the ventral part of the pars triangularis: vIFG, 51 32 1 and one in the dorsal part of the pars triangularis: dIFG, 45; 20; 19) responded more to the observation of ToM videos than to the observation of RANDOM video clips. See Table 2 for an overview of the brain activation related to the ToM > RANDOM contrast and the accompanying statistics.

**Table 2:** MNI coordinates of peak activations during the localizer task associated with the contrast ToM > RANDOM. Reported clusters are significant at *p* < 0.05 (FWE corrected) after applying a whole brain uncorrected threshold of *p* < 0.001 at voxel level.

	Peak coordinate (MNI)	Z-score	Cluster Size (k)
**ToM > RANDOM**			
Left superior temporal sulcus (lSTS)	-60–55 7	5.53	423
Right superior temporal sulcus extending into the right temporoparietal junction (rTPJ)	57–55 1	5.11	1348
Right inferior frontal gyrus, ventral part of the pars triangularis (vIFG)	51 32 1	4.74	111
Right inferior frontal gyrus, dorsal part of the pars triangularis (dIFG)	45 20 19	3.74	93

### Passive task

**Fig 1:**
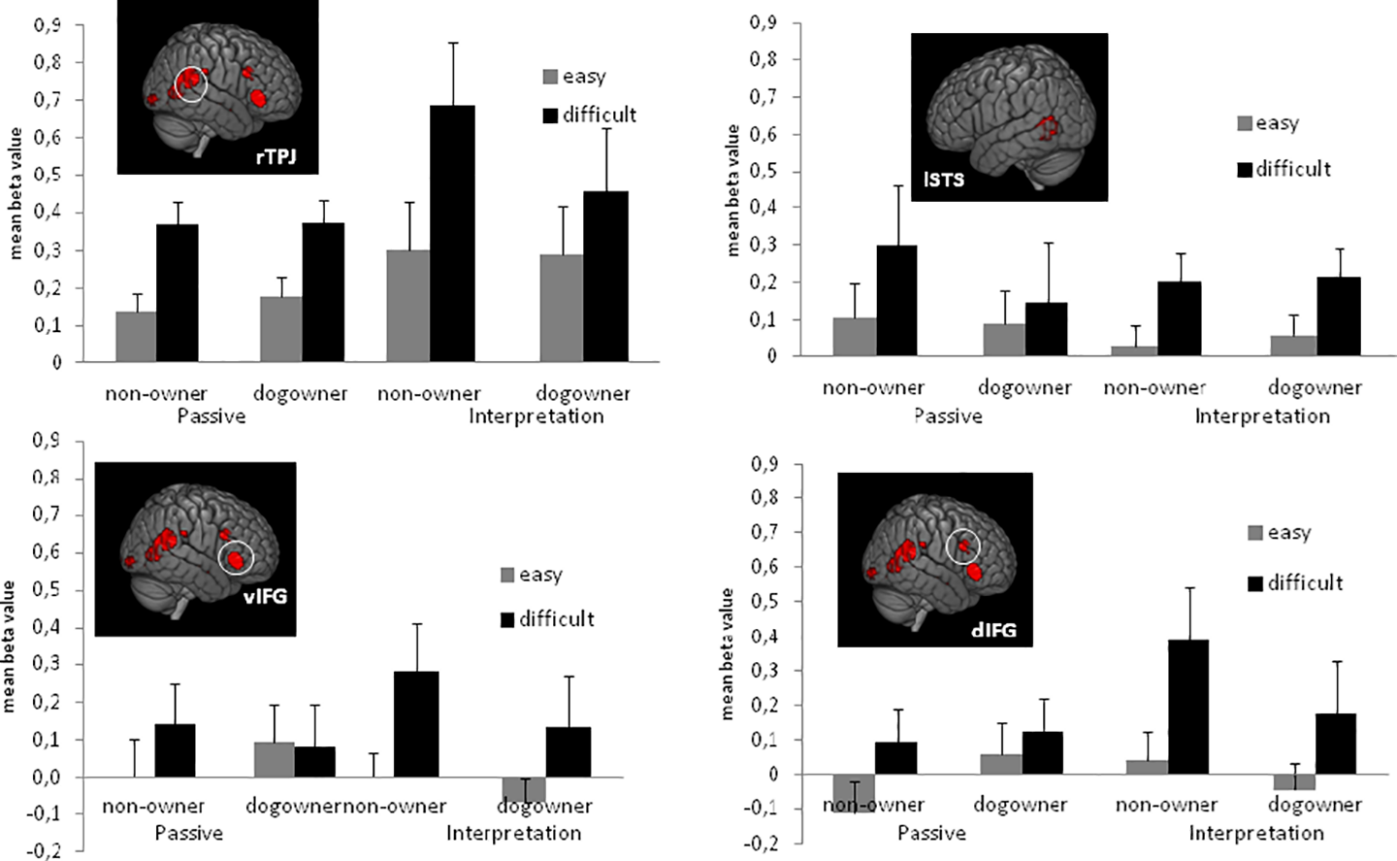
Mean beta values for easy and difficult trials in the rTPJ (57–55 1), lSTS (-60–55 7), vIFG (51 32 1) and dIFG (45 20 19) during the passive and the interpretation task for participants who do not own a dog and participants who own a dog. Brain activations are taken from the localizer task at *p* < 0.001 uncorrected. To correct for multiple comparisons a cluster-level extent threshold was applied at *p* < 0.05 (FWE cluster corrected threshold).

Three out of the four ROI's showed a main effect of DIFFICULTY. More precisely, the lSTS (*F*(1,15) = 9.53, *p* < 0.01), the rTPJ, (*F*(1,15) = 38.54, *p* < 0.001), and the dIFG (*F*(1,15) = 6.49, *p* < 0.05) all showed larger beta-values during the observation of the difficult video clips compared to the observation of the easy video clips (for the vIFG: *F* < 1). Neither the main effect of DOG OWNER nor the interaction of DOG OWNER x DIFFICULTY reached significance in any of the ROI's (Main effect for DOG OWNER: dIFG: *F*(1,15) = 1.74, *p* = 0.21, for the three other ROI's: *F*s < 1. Interaction between DOG OWNER and DIFFICULTY: vIFG: *F*(1,15) = 1.39, *p* = 0.26, dIFG: *F*(1,15) = 1.76, *p* = 0.21, for the lSTS and the rTPJ, *F*s < 1.) See Fig 1 for a plot of the results and S1 Appendix for a complete overview of the mean beta values.

### Interpretation task

A main effect of DIFFICULTY was observed for all ROI's (lSTS: *F*(1,15) = 6.62, *p* < 0.05, rTPJ: *F*(1,15) = 29.01, *p* < 0.001, vIFG: *F*(1,15) = 9.88, *p* < 0.01, dIFG: *F*(1,15) = 19.69, *p* < 0.001). As in the PASSIVE task, beta-values were larger during the observation of the difficult video clips than during the observation of the easy video clips. None of the ROI's showed a significant main effect of DOG OWNER (vIFG: *F*(1,15) = 2.23, *p* = 0.16, dIFG: *F*(1,15) = 2.13, *p* = 0.17, for the lSTS and rTPJ, *F* < 1). Interestingly, the interaction between DOG OWNER and DIFFICULTY was marginally significant for the rTPJ (*F*(1,15) = 4.44, *p* = 0.05). This interaction did not reach significance for the other ROI's (lSTS: *F*(1,15) = 1.89, *p* = 0.19, for the vIFG and the dIFG *F*s < 1). See Fig 1 for a plot of the results and S1 Appendix for a complete overview of the mean beta values.

## General discussion

In the current fMRI study we investigated to what extent brain regions associated with mentalizing are engaged when observing dog behaviour that is difficult to interpret compared with dog behaviour that is easy to interpret. Further, we examined if this pattern could be observed both when participants passively observed the behaviour and when they actively inferred reasons for the dog behaviour. Finally, we investigated if dog ownership modulated these effects.

To identify regions associated with interpreting behaviour all participants completed an independent localizer task [19]. Four regions were defined as ‘mentalizing regions’ based on this localizer task: lSTS, rSTS extending into the rTPJ, vIFG, and dIFG. The rTPJ is considered to be the core region of the ToM network [20]. The engagement of STS and IFG have been reported when using ToM paradigms in fMRI studies but these regions are not necessarily considered as core ToM regions (for a review see [21]). In the last part of the discussion we will come back to this issue. In the reminder of this manuscript we will refer to these four regions as ‘mentalizing regions’. We will first elaborate further on the four main conclusions of this study.

### Trying to interpret behaviour and the ToM network

First of all, the mentalizing regions (rTPJ, lSTS, vIFG, dIFG) were more engaged during the observation of dog behaviour that is difficult to interpret compared to the observation of dog behaviour that is easy to interpret. Previous studies already found this network to be stronger engaged when observing human behaviour that is difficult to interpret in comparison with human behaviour that is more easy to interpret (e.g. [8, 12]). This suggests that this network is related to the amount of effort humans have to put into interpreting a behaviour: a more difficult behaviour engages the network more strongly. Here, we show that this not only applies when trying to interpret human behaviour but also when trying to interpret non-human behaviour. According to [22], humans will process “the content of mind of the subject” once agency is detected. In accordance with this idea, we show here that the processes underlying the ToM network are not specifically related to trying to interpret human behaviour but are more general. Probably, the observation of an intentional agent (a dog) is sufficient to engage this system.

### Passive observation and activation of the ToM network

Second, and in line with studies on the observation of human agents, the difference in engagement between EASY and DIFFICULT video clips was present in both tasks (PASSIVE and INTERPRETATION). It thus seems that the mentalizing network is not only engaged when humans actively try to infer reasons for the dog behaviour but that it is also more engaged when humans merely observe dog behaviour that is more difficult to interpret. This finding is in line with fMRI studies on the observation of human behaviour (e.g. [12, 13]). More generally, this finding parallels observations obtained with other typical theory of mind tasks such as perspective taking or false belief tasks (e.g. [23]). When assessing ToM via the use of perspective taking or false belief tasks, it has been shown that humans not only take into account others' perspectives when they consciously want to, but that they spontaneously take perspective, even if this is not beneficial [24]. The data here show that when humans passively observe dog behaviour, there is a difference in engagement in the mentalizing network between difficult and easy interpretable dog behaviour. This suggests that the processes associated with the interpretation of behaviour are spontaneous in the sense that they are not explicitly required by the instructed task. This finding might be important for further research on human-animal interactions because it hints at spontaneous processing of dog behaviour (and probably animals in general). This suggest that we process animal behaviour in the same way as we process human behaviour. Further research is needed here but it might relate to other findings concerning human-animal interactions. For example, the fact that humans show empathy for other species [25] and why animal welfare is such a largely discussed topic in our current society. Furthermore, this might to some degree explain why animals sometimes strongly impact the mental health of people that are socially isolated. In such situations a dog might trigger similar social processes as a human interaction partner.

In the current study we decided to always present the PASSIVE task first. We choose for this solution because presenting the task with the instructed condition first (i.e., the INTERPRETATION TASK) would presumably have had carry over effects on the task without the instructed condition (i.e., the PASSIVE TASK). However, this is an unavoidable limitation of the current study and needs to be taken into account when interpreting the results.

### Spontaneous interpretation and dog ownership

Interestingly, the engagement of the mentalizing network during the PASSIVE task did not differ significantly between dog owners and non-owners. Intuitively, one could think that dog owners automatically start to infer reasons for dog behaviour when they observe a dog because they are intrinsically motivated to understand dogs, whereas non-owners would not feel this need and therefore would not infer potential reasons for the behaviour. If this was true, one would have expected a larger engagement in the mentalizing network during the PASSIVE task for owners compared to non-owners. However, our data are not in line with this prediction. Further research should clarify this issue more. More extreme groups could be studied to answer this question. For example, a stronger engagement of the mentalizing network in the PASSIVE task could be obtained for dog owners compared to no non-owners if dog owners would observe their own dogs. On the other hand, more extreme groups of non-owners, such as people that have fear of dogs, could show no differences in engagement between easy and difficult dog behaviour during the PASSIVE task.

Also the way “dog expertise” is measured seems to be crucial. In this study we classified subjects as dog owners and non-owners. However, Schirmer and colleagues [26] showed that the frequency of interaction with dogs seems more relevant than ownership to predict emotion ratings of dogs. In this study we did not assess frequency of interaction, but further research should take this into account. Other research investigating the role of experience when interpreting dog emotions, indicates that experience is related to interpreting fearful behaviour of dogs but not happy behaviour of dogs [27]. So the role of experience might only be reserved for trying to interpret specific emotions or specific dog behaviour.

### Dog ownership and the rTPJ

Our fourth and final conclusion concerns the role of experience with dogs on the engagement of the rTPJ. This finding should be interpreted with caution since it was only marginally significant (*p* = 0.05) in one out of several tests conducted. However, because the right temporo-parietal junction (rTPJ) has been pinpointed as the key brain area in theory of mind [19, 28, 29] we would like to elaborate a bit further on this finding. Our results indicate that the difference in brain activation when observing EASY and DIFFICULT video clips was less pronounced for dog owners than for non-owners during the INTERPRETATION task in the rTPJ. Closer inspection of the data showed that this smaller difference was caused by a reduction in activation during the observation of difficult video clips for dog owners compared to non-owners. Two hypotheses concerning the influence of experience in this ToM area can be proposed. First, one could hypothesize that dog owners will show a stronger engagement of this area when they observe behaviour of dogs because they will more heavily anthropomorphize dogs [30]. On the other hand, one can argue that dog owners will show a smaller response in regions associated with ToM processes when trying to interpret dog behaviour because the experience they possess with dog behaviour will make the interpretation of the behaviour easier. Research has indeed shown that the interpretation of dog behaviour improves with the amount of experience humans have with dogs. Lakestani and Donaldson [31] for instance, found that recognition of dog behaviour (for example aggressive behaviour) improved after a short training program. Further, Schirmer and colleagues [26] showed that the frequency with which participants interacted with dogs influenced the recognition of dog affect (see also [32, 33]).

Our data are in line with this second hypothesis: it seems that the rTPJ is more responsive when humans are instructed to interpret behaviour with which they have less experience (dog behaviour for a non-owner). As previously mentioned this interaction did not reach significance. Therefore, this finding should be interpreted with caution and further research is needed to confirm this interaction.

In line with our suggestion, Saxe and Wexler [20] found that the response in the posterior and dorsal part of this area was higher when participants had to read mental states of persons with a foreign background compared to reading mental states of people with a familiar background. These authors concluded that the rTPJ is the crucial node in the ToM network and is assumed to be responsive to the attribution of mental states [20]. We must mention here that the area that we labeled as rTPJ extends into the rSTS and is thus more anteriorly and more dorsally located than the location labeled as rTPJ by Saxe and colleagues [20, 28, 29]. One could think that the difference in rTPJ engagement between owners and non-owners relates to better mentalizing abilities for dog owners. However, earlier research indicated that dog ownership is not related to more mentalizing abilities in general. No differences were found on empathy measures between dog experts and non-experts [3]. However, dog experts were better in visually reading the body postures of dogs compared to non-experts. It seemed that dog-experts look more to the bodies than to the heads compared to non-experts [3]. In line with these findings our result suggests that the reduced engagement of the rTPJ for dog owners when trying to interpret DIFFICULT dog behaviour is related to a different -or more efficient- processing of the dog behaviour for dog owners compared to non-owners.

### ToM network

As mentioned above, our independent localizer identified four regions that showed higher activation during the observation of the ToM video clips compared to the RANDOM video clips in the localizer task. These regions were the rTPJ, the lSTS, and two regions (one in the ventral part and one in the dorsal part) in the IFG (vIFG, dIFG). As already mentioned previously, the rTPJ is considered the core region of the ToM network [20]. IFG and STS, although not part of the typical ToM network, have been related to ToM processing before. Moreover, the STS has been related to the processing of biological motion (e.g. [34]). As a result, the functional role of the STS in ToM might be the use of motion cues to interpret behaviour (e.g. [35]). IFG activation, has been reported in different ToM paradigms and has been related to the inhibition of self-perspective [36]. Surprisingly, we did not observe medial prefrontal activity when comparing ToM video clips with RANDOM video clips. However, other studies did not obtain medial prefrontal activation either when using social animation tasks. In fact, the meta-analysis of Schurz and colleagues [37] shows that the mPFC activation when using social animation tasks is only reproduced in 8 out of the 14 studies. According to these authors, the mPFC is primarily important when judging characteristics of another person, such as for example a personality trait (see also [38]). In line with this, the mPFC has been argued to serve a broader function in social cognition and thus be less crucial when reasoning about other's behaviour [29].

## Conclusion

In this study we show that lSTS, rTPJ/STS, and IFG are more responsive to dog behaviour that is difficult to interpret compared to dog behaviour that is easy to interpret. This is the case under explicit instructions to infer reasons for the observed behaviour and under passive observation of the behaviour. While dog ownership did not yield a statistically robust influence on the involvement of the mentalizing system when observing dog behavior, a statistical trend in the rTPJ was observed when participants were explicitly instructed to infer the underlying reasons of the dog behaviour. On a descriptive level, dog ownership did lead to a reduced difference in response in interpreting easy and difficult dog behaviour. However, this latter finding can only be seen as a first indication for such an influence since the result was only marginally significant and not corrected for multiple comparisons.

## Supporting information

S1 Appendix Mean beta values associated with the fMRI task analyses. Columns A till Q represent respectively dog ownership (1 = dog owner), the mean beta values associated with the passive task and the interpretation task for the easy and the difficult video clips extracted in the four regions taken from the localizer task (rTPJ, lSTS, dIFG and vIFG).
(XLSX)
